# Prediction of Protein-Protein Interactions from Amino Acid Sequences Based on Continuous and Discrete Wavelet Transform Features

**DOI:** 10.3390/molecules23040823

**Published:** 2018-04-04

**Authors:** Tao Wang, Liping Li, Yu-An Huang, Hui Zhang, Yahong Ma, Xing Zhou

**Affiliations:** Department of Information Engineering, Xijing University, Xi’an 710123, China; wangtao@xijing.edu.cn (T.W.); cs2bioinformatics@gmail.com (L.L.); 20040020@xijing.edu.cn (H.Z.); mayahong@xijing.edu.cn (Y.M.); xingzhuo@xijing.edu.cn (X.Z.)

**Keywords:** protein-protein interaction, protein sequence, weighted sparse representation

## Abstract

Protein-protein interactions (PPIs) play important roles in various aspects of the structural and functional organization of cells; thus, detecting PPIs is one of the most important issues in current molecular biology. Although much effort has been devoted to using high-throughput techniques to identify protein-protein interactions, the experimental methods are both time-consuming and costly. In addition, they yield high rates of false positive and false negative results. In addition, most of the proposed computational methods are limited in information about protein homology or the interaction marks of the protein partners. In this paper, we report a computational method only using the information from protein sequences. The main improvements come from novel protein sequence representation by combing the continuous and discrete wavelet transforms and from adopting weighted sparse representation-based classifier (WSRC). The proposed method was used to predict PPIs from three different datasets: yeast, human and *H. pylori*. In addition, we employed the prediction model trained on the PPIs dataset of yeast to predict the PPIs of six datasets of other species. To further evaluate the performance of the prediction model, we compared WSRC with the state-of-the-art support vector machine classifier. When predicting PPIs of yeast, humans and *H. pylori* dataset, we obtained high average prediction accuracies of 97.38%, 98.92% and 93.93% respectively. In the cross-species experiments, most of the prediction accuracies are over 94%. These promising results show that the proposed method is indeed capable of obtaining higher performance in PPIs detection.

## 1. Introduction

Participating in almost every aspect of cellular function within an organism, proteins are the work-horses of the cellular machinery. Usually, they cooperate with each other, forming a big interaction network rather than carrying out particular biological functions alone. The prediction of protein-protein interactions has comes to be a hot spot in studies on proteomics. Research has shown that proteins with similar functions are more likely to interact. With a protein of unknown function, it is feasible to predict its function based on its binding partners. Therefore, predicting interactions of protein pairs helps to understand the functional roles of unannotated proteins. In addition, abnormal protein-protein interactions (PPIs) which lose their function or stabilize at an inappropriate time or location are associated with many diseases, such as autoimmune diseases and cancer. For this reason, predicting PPIs can provide great insight for designing drugs. It is estimated that there are as many as 650,000 different PPIs which compose the whole human protein-protein interactome and discovering them would be a long-term task. All these factors collectively have led to increased attention on predicting PPIs [1].

Much effort has been devoted to developing experimental techniques for finding protein-protein interactions, such as yeast two-hybrid (Y2H) [2,3] screens, tandem affinity purification (TAP) [4], mass spectrometric protein complex identification (MS-PCI) [5] and other high-throughput biological techniques for PPIs detection. As obtained from these biological experiments, protein interaction data have been stored by a number of constructed databases, such as MINT [6], BIND [5] and DIP [7]. However, these experimental methods are tedious and cost a lot. The PPIs identified by them only cover a little fraction of the whole PPI network. In addition, the experimental methods are usually associated with high rates of both false positive and false negative predictions. All of these drawbacks have stimulated research on computational methods for predicting PPIs.

Different types of protein data have been obtained by previous experimental methods, including protein sequences, secondary structures and tertiary structures. In order to utilize this wealth of protein data, a number of computational approaches have been proposed. Among them, it is popular to predict PPIs based on protein structure data. For example, Agrawal et al. [8] proposed a computational tool named the spatial-interaction-map (SIM) which utilizes the structure of unbound proteins to predict the residues of protein-protein interactions. Qiu et al. [9] presented a novel residue characterization model based on 3D structure with the purpose of detecting PPIs. These computational methods based on structural data identify the interaction domain by analyzing the hydrophobicity, solvation, protrusion and the accessibility of residues. Since the amount of newly discovered protein sequence data is increasing exponentially, there is an increasingly larger gap between the amount of protein structure data and that of protein sequence data. Predicting PPIs based on structural data cannot satisfy requests of the great number of biochemists, most of whom obtain protein sequences, but no structural data. Therefore, it is more important to develop an effective computational model based on protein sequences [10,11].

Up until now, a number of sequence-based computational methods have been proposed. Most of them utilize information regarding protein homology or the interaction marks of the protein partners. In related works, Zahiri [12] proposed a method with a novel protein evolutional feature extracted from position-specific scoring matrix (PSSM)s for predicting protein-protein interactions. However, it is becoming be more and more difficult to use sequence homology recognition approaches to predict PPIs due to the decreased similarity between proteins and their homologues. For this reason, it is more practical to predict PPIs using only the information from protein sequences.

Generally, computational models for PPI predictions are composed of two parts: feature extraction and sample classification. As the first step, feature extraction aims to represent proteins with useful attributes and transform the samples into feature vectors of the same size as the inputs of the sample classifier. Effective feature descriptors can play efficient roles in improving the prediction performance of the system. In this work, we adopt a novel feature extraction method using continuous and discrete wavelet transforms. Specifically, as wavelet transform can only deal with numerical signals, we first transform every protein sequence into a real sequence by substituting each amino acid character with a specific corresponding physicochemical property, and here, we choose the hydrophobicity index for this transformation. Hydrophobicity is known to be important for protein interaction as it is associated with protein folding and unfolding [13]. Next, we represent a protein as a 60 × 60 continuous wavelet (CW) image with continuous wavelet transform and then utilize the singular value decomposition (SVD) method to extract CW-SVD descriptors from the CW images. In addition, we execute two-scale biorthogonal discrete wavelet (DW) transform on every protein’s real sequence and then form the DW descriptors with the first five discrete cosine coefficients from the approximation coefficients and the maximum, minimum, mean and standard deviation values from both the detail and approximation coefficients. 

Most of the proposed computational prediction models for PPIs adopt traditional classifiers based on machine learning approaches, such as the support vector machine (SVM) [14,15,16] and neural network (NN) [17]. Although these traditional classifiers have proven powerful for classification, they usually need much labor and time to search the optimal values of parameters for the best performance. Recently, the sparse representation-based classifier (SRC) has been earning a reputation for its outstanding performance in the field of signal processing, pattern recognition and computer vision. In this work, we explored the weighted sparse representation-based classifier (WSRC), a variant of basic SRC, for predicting protein-protein interactions. WSRC integrates both sparsity and locality structure data into the SRC and therefore, surpasses conventional SRC.

In this work, we present a computational model for predicting protein-protein interactions only using the information from amino acid sequences. This model combines the sparse representation-based classifier and a novel representation method composed of CW-SVD features and DW features. We first searched the best scale value of discrete wavelet transform applied in protein sequences and then extracted features from protein sequences using continuous and discrete wavelet transforms. Each protein was finally represented by an 86-dimensional feature vector, consisting of the 60-dimensional CW-SVD descriptor and the 26-dimensional DW descriptor. WSRC was adopted in the final step. We explored the proposed method for the prediction of PPI data from three different biological datasets: yeast, humans and *H. pylori*. For further evaluation of the performance, the method based on SVM was also compared with the proposed method. In addition, we applied the proposed method to predict protein-protein interactions of other species using the data from yeast. Specifically, we used the whole yeast dataset as a training set and the six other species’ datasets as testing sets.

## 2. Results

### 2.1. Parameter Selection

In this work, the two corresponding parameters, *σ* and *ε*, were set to be 1.5 and 0.00005, respectively, when using the weighted sparse representation-based classifier. Since the scale of discrete wavelet transform is the unique parameter for the feature extraction method, the selected value of scale would influence the efficiency of the whole feature extraction. For this reason, we explored the proposed method with different scale values to search for the optimal value for the best performance. We implemented a series of experiment for parameter adjustment and found that the performance maintains a slightly decreasing trend when the scale factor is adjusted from 2 to 10. This may be rooted in the fact that a high scale increases the computational complexity and decreases the accuracy. Finally, we set the scale parameter for the discrete wavelet transform used in feature extraction as 2.

### 2.2. Assessment of Prediction Ability

**Figure 1:**
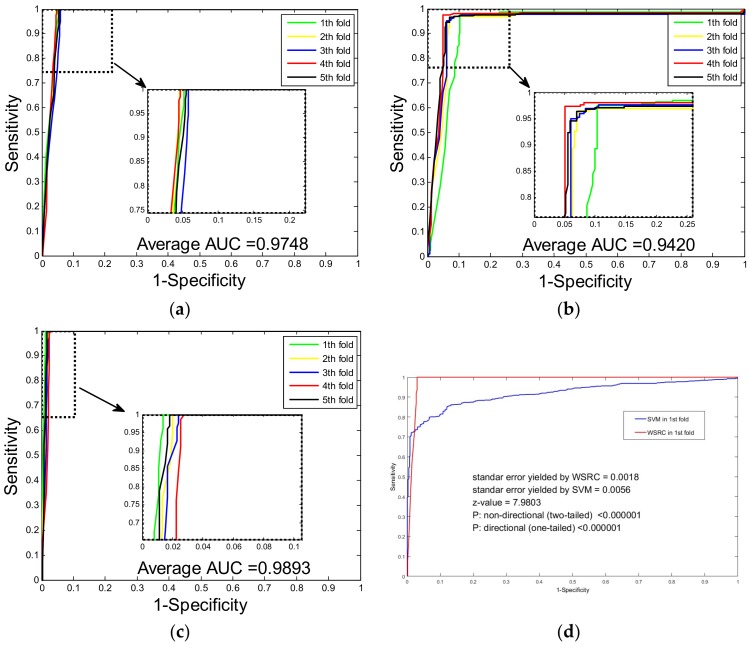
ROC cures yielded by five-fold cross validation: (**a**) ROC from proposed method result for yeast protein-protein interactions (PPIs) dataset; (**b**) ROC from the proposed method result for *H. pylori* PPIs dataset; (**c**) ROC from proposed method result for Human PPIs dataset; (**d**) comparison of ROCs between the weighted sparse representation-based classifier (WSRC) method and the support vector machine (SVM) method on the first fold of Human PPIs dataset.

For the sake of fairness, in this work, we set the same corresponding parameters of weighted sparse representation-based classifier when predicting PPIs of the yeast and *H. pylori* datasets. It is common to use five-fold cross validation to evaluate the fit of the proposed model to the hypothetical validation. In this work, we used this cross-validation method to avoid overfitting and to evaluate the performance stability of the proposed method. Specifically, we experimented on one dataset over five rounds of cross-validation. The whole dataset was partitioned into five parts, where four parts are used for training and the other part was used for testing. The prediction results performed by the proposed method on the yeast dataset are shown in Table 1. It can be observed that when using the proposed method to predict PPIs of the yeast dataset, we obtained promising results with averages of accuracy, precision and sensitivity as high as 97.38%, 100.00% and 94.76%, respectively. From Table 2, which shows the five-fold cross validation results of the *H. pylori* dataset, we can see that the proposed method yielded good results with averages of accuracy, precision and sensitivity as high as 93.93%, 96.41% and 91.20%. In addition, it should be noticed that the standard deviations of the criteria are relatively low. In the experiment involving the yeast dataset, the standard deviations of accuracy, precision and sensitivity were 0.31%, 0.00% and 0.68%. When predicting PPIs in the *H. pylori* dataset, the standard deviations of accuracy, precision and sensitivity were 1.11%, 0.81% and 2.15% respectively. To allow a more comprehensive assessment, the corresponding MCC (Matthews correlation coefficient) values and AUC (the area under an ROC curve) scores of the experiments were also computed. When predicting the PPIs of the yeast dataset, the average MCC and AUC achieved values of 94.89% and 97.48%, with standard deviations of 0.59% and 0.26% (see Figure 1a,b). When exploring the *H. pylori* dataset, the average MCC and AUC values were 88.57% and 94.20%, and the corresponding standard deviations were 1.95% and 1.05%.

### 2.3. Comparison with SVM and Single Wavelet Feature Descriptor

A wide range of machine learning prediction models have been proposed for the field of protein interaction prediction, and one of the most popular classifiers is SVM. In this section, using the same feature extraction method, we compare the prediction performance of the proposed method with the SVM-based method on the dataset of humans. To obtain optimal parameters for the SVM classifiers, c and g, a grid search method was used. Here, we set c = 0.8 and g = 0.7 by using the grid search method. Table 3 shows the comparison results of the WSRC and SVM classifiers, and we can see that when using the proposed method to predict the PPIs in the human dataset, the averages of accuracy, precision and sensitivity are as high as 98.92%, 99.95% and 97.77%, respectively. However, when exploring the human dataset with the SVM-based method, we gained relatively low averages of accuracy, precision and sensitivity of 90.13%, 96.14% and 82.41%, respectively. In addition, the standard deviations of these criteria performed by the proposed method were lower than the standard deviations yielded by the SVM-based method. When using the WSRC-based method to explore the human dataset, the standard deviations of accuracy, precision and sensitivity were 0.27%, 0.07% and 0.57% while the standard deviations performed by SVM classifier were 1.22%, 1.00% and 1.85%. The MCC values and AUC scores for the comparison experiment were also computed. The ROC curves of the comparison experiment are shown in Figure 1c,d. It can be observed that the WSRC yielded average MCC and AUC values as high as 97.86% and 98.93%, with corresponding standard deviations of 0.52% and 0.37%. However, the SVM-based method yielded relatively poor results, with an average MCC value of 81.87% and an average AUC score of 93.99%, with standard deviations of 1.99% and 1.52%. Considering the higher values for criteria and lower standard deviations, WSRC is superior to the SVM classifier with higher accuracy and better stability. In addition, we also did comparison experiments to analyze the performance between using CW-SVD alone and using the DW feature descriptor alone. When combined with WSRC and performed on the human dataset, CW-SVD yielded average accuracies of 96.85 and 98.71, respectively, lower than that yielded by their combination: 98.92. The result demonstrates the effectiveness of their combination for protein sequence feature extraction.

### 2.4. Comparison with Other Methods

Various kinds of computational methods have been proposed for predicting protein interactions. In this section, we compare the prediction performance between the proposed method and other different existing methods on the yeast and *H. pylori* datasets.

Table 4 shows the cross-validation results performed by other existing methods on the yeast dataset, and it can be observed that the average accuracies yielded by these methods are between 75.08% and 92.05%, lower than the 97.38% accuracy which was obtained by the proposed method. Considering the precision and sensitivity, none of the other methods can perform better than the proposed method which yielded the highest precision and sensitivity values of 100.00% and 94.76%, respectively. In addition, compared with the other methods, the proposed method yielded relatively low standard deviations foraccuracy, precision and sensitivity of 0.31%, 0.00% and 0.68%, respectively. In addition, we also compared the proposed combined wavelet feature descriptor with some other previously proposed methods. When combined with the same classifier as WSRC, these proposed feature extraction methods yielded prediction accuracies ranging from 96.03 to 96.60, lower than that of our method. When introducing the pseudo amino acid descriptor into the combined wavelet-based feature, the model obtained a poorer performance with an average accuracy of 0.9731 ± 0.003. Table 5 shows the average results performed by other existing method on the *H. pylori* dataset, and from that, we can see that the accuracy yielded by the proposed method (93.93%), is the highest among all six methods. In addition, compared with the other methods, our proposed method yielded the higher average precision and sensitivity values of 96.41% and 91.20%.

### 2.5. Performance on Independent Dataset

Since the proposed model gave a good performance when predicting PPIs on the three datasets, to further evaluate the generalization ability, we used the whole samples from the yeast dataset as the training set to predict the PPIs of six other species: *D. mela*, *E. coli*, *C. elegans*, *H. sapien*, *H. pylori* and *M. musculus*. For these six datasets, all the samples were positive. From Table 6, it can be observed that when predicting the PPIs of *D. mela*, *E. coli*, *C. elegans*, *H. sapien*, *H. pylori* and *M. musculus*, the proposed method yielded excellent results with accuracies as high as 97.36%, 86.56%, 96.64%, 94.24%, 95.07% and 94.23%, respectively. For these experiments, most of prediction accuracies were over 94% and the highest accuracy even reached 97.36%. When predicting the PPIs on the *E. coli* dataset, we obtained the lowest prediction accuracy, but this was still over 86.5%. Interestingly, these outstanding results show that it is sufficient to use yeast PPIs data to predict the PPIs of other species. Our proposed method is powerful enough to deal with cross-species PPIs prediction and has excellent generalization ability which may offer experience for further research.

## 3. Discussion

Continuous wavelet transform (CWT) and district wavelet transform (DWT) are two useful and popular transformations used in various fields, and they are mutually complementary. In this work, the outstanding performance yielded by our method on three standard PPI datasets shows the feasibility and effectiveness of the proposed feature extraction method which combines the CWT-SVD and DWT descriptors, and the low standard deviations show that our proposed prediction method for protein interactions is robust. The effectiveness of the combined wavelet features may come from the novelty of transforming the protein sequences into images (matrixes) and using the wavelet transformation to extract the feature vectors. The physicochemical properties (hydrophobicity, in this work) could be well embedded in this transformation. In addition, the comparison between combined wavelet feature and single wavelet feature demonstrates that these two features may be complementary to each other and can further enhance the representation ability of a single type of feature.

There are some possible reasons accounting for the good results yielded by our proposed method. One reason lies in the fact that the sparse representation-based classifier performs well with matrix features, such as CWT features, which are derived from primary sequences. As we transformed the protein sequences into matrixes by using the hydrophobicity index, we can regard the matrixes as images and use the classifier in the field of image processing to classify the samples. WSRC integrates both sparsity and locality structure data into conventional SRC and therefore, further improves the classification performance. Besides, when using the WSRC, little manual intervention is needed to adjust the corresponding parameters, helping us to obtain good results without much effort. WSRC tries to use a linear combination of training samples to reconstruct a testing sample. Each training sample would be assigned a weight for this combination in the learning process, and the samples with greater weights would play more important roles in the prediction. For example, in the task of image classification, images from the sample label may contain similar textures, and SRC would assign these images with greater weights for prediction. This algorithm principle is in accordance with the fact that proteins of similar structure and physicochemical properties tend to have common interaction partners. In addition, the sizes of the datasets that we explored in this work were relatively small (about 10,000 or less) compared with the other prediction problems involved in machine learning. As SRC are known to be effective on small and midsize datasets, datasets of this size could be good choices in the construction of a prediction model for PPI interactions.

It is known that methods which are based on an ensemble classifier usually achieve more accurate and robust performances than methods which use a single classifier. However, even though the weighted sparse representation-based classifier is a single classifier, its performance is superior to some other methods which use ensemble classifiers, such as boosting and ensemble of HKNN. The results of comparison experiments applied to the yeast and *H. pylori* datasets demonstrate that the proposed method combing continuous and discrete wavelet transform features can improve the prediction accuracy.

## 4. Materials and Methods

### 4.1. Datasets

In this work, the proposed method was verified with a high confidence Saccharomyces cerevisiae PPIs data set. We collected this dataset from publicly available database of interacting proteins (DIP). Protein pairs with ≥40% sequence identity or whose lengths were less than 50 residues were removed. By doing this, we obtained the remaining 5594 protein pairs which were further used to construct the positive data set. For the negative dataset, we used 5594 additional protein pairs of different sub-cellular localizations. As a result, the whole data set was finally made up of 11,188 protein pairs, of which half were from positive samples and half were from negative samples.

To demonstrate the generality of the proposed method, we also verified our approach on two other types of PPIs data sets. The first dataset was collected from the Human Protein References Database (HPRD). Protein pairs with ≥25% sequence identity were removed. Finally, to comprise the golden standard positive dataset, we used the remaining 3899 protein-protein pairs of experimentally verified PPIs from 2502 different human proteins. For the golden standard negative dataset, we followed previous work [29] and assumed that the proteins in different subcellular compartments do not interact with each other. Specifically, the negative dataset was randomly generated from the Swiss-Prot database (version 57.3) by excluding protein sequences which met the following conditions: (i) protein sequences without a certain subcellular location; (ii) protein sequences annotated with more than one subcellular location or “fragment” term; (iii) protein sequences of less than 50 amino acids. Finally, we obtained 4262 protein pairs from 661 different human proteins and used them to construct the negative dataset. As a result, the human dataset contained 8161 protein pairs. The second PPI dataset contained 2916 Helicobacter pylori protein pairs, of which 1458 were interacting pairs and 1458 were non-interacting pairs, as described by Martin et al.

### 4.2. Continuous Wavelet Transformation

It is popular to use wavelet transform to extract information from many different kinds of data as a mathematical tool. In the studies involving the prediction of PPIs, Li et al. [30] suggested using wavelets as descriptors to represent proteins and proved that it is feasible to use wavelet transform in feature extraction for proteins. Compared with Fourier transform, wavelet transform has a completely different merit function. It uses functions which are localized in both the real and Fourier space, while Fourier transform decomposes the input signal into sines and cosines. As an implementation of the wavelet transform, continuous wavelet transform (CWT) uses arbitrary scales and almost arbitrary wavelets. The child wavelets of CWT can be symbolized as follows:

(1) $$\psi_{a,b}(t)=\frac{1}{\sqrt{a}}\psi (\frac{t-b}{a})$$

where *a* denotes the scale factor and *b* denotes the shift scale. Based on the child wavelets, the subspace of scale *a* is generated. The continuous wavelet transform can be further defined as follows:

(2) $$CWT_{f}(a,b)=\langle f,\psi_{a,b}\rangle =\frac{1}{\sqrt{a}}\int f(t)\psi (\frac{t-b}{a})dt$$

where *f*(*t*) is the digital signal sequence. Continuous wavelets are localized in both time and frequency domains. In addition, since it reinforces the traits due to the redundancy tends, continuous analysis is often easier to interpret. In this study, we applied Meyer continuous wavelet transform with 60 decomposition scales to transform every protein numerical sequence into a 60 × 60 matrix. Singular value decomposition was then used to extract a 60-dimensional vector from each CW matrix, which constructed the CW-SVD descriptor for the protein sequences.

### 4.3. Discrete Wavelet Transform

Discrete Wavelet Transform (DWT) is another implementation of the wavelet transform. Different from continuous wavelet transform, DWT uses a discrete set of the wavelet scales and translations and decomposes the input signal into a mutually orthogonal set of wavelets. It is common to use discrete wavelet to denoise a noisy signal. Similar to CWT, DWT can be expressed as

(3) $${\mathrm{DWT}}_{f}(a,b)=\frac{1}{\sqrt{a}}\int f(t)\psi (\frac{t-b}{a})dt$$

where *a* and *b* are the scale and shift parameters and belong to a set of real numbers. *a*_0_ and *b*_0_ are set to be 2 and 1 respectively. Hence, the results can lead to a binary dilation of 2^−*p*^ and a dyadic translation of *q*2*^p^*. The wavelet core of DWT can be described as

(4) $$\psi_{p,q}(x)=2^{-p/2}\psi (2^{-p}x-q)$$

where *p* = 1, 2, … and *q* = 0, 1, 2, … Therefore, DWT can further be described as

(5) $${\mathrm{DWT}}_{f}(a,b)=\langle f(x),\psi_{a,b}(x)\rangle =2^{-p/2}\int f(x)\psi (2^{-p}\cdot x-q)dx$$

The coefficients of the DWT are composed of two parts: approximation and detail coefficients. Approximation coefficients account for the high-scale and low-frequency components of input signals while detail coefficients represent the low-scale and high-frequency components. In this work, we first transformed protein sequences into numerical sequences based on the hydrophobicity index and then optimized the scale parameter of DWT to give the best performance. Since high-frequency components contain more noise, we tended to choose approximation coefficients to represent protein sequences. For this reason, we finally chose the first five discrete cosine coefficients from the approximation coefficients and the maximum, minimum, mean and standard deviation values of both approximation and detail coefficients to construct the 26 DW descriptors for the protein sequences. Then, all descriptors from the CW-SVD and DW descriptors were concatenated and a final 86-dimensional vector was built to represent each protein sequence. Finally, each PPI pair was characterized by concatenating the two vector spaces of two individual proteins. Thus, a 172-dimentional vector was been constructed to represent each protein pair and was used as a feature vector for input into the classifier.

### 4.4. Weighted Sparse Representation Based Classifier

With the great progress in the compressed sensing (CS) and the linear representation methods (LRBM), sparse representation has been gradually developed for prediction classification. In particular, in the field of signal processing, computer vision and pattern recognition, the sparse representation based classifier (SRC) [31] plays a powerful role and has earnt a reputation for its strong ability to cope with illumination variations, occlusions, and random noise. Sparse representation tries to optimize a matrix to reveal the relationship between any given test sample and the training set. Through this matrix, a sample can be represented by the linear combination of training samples, and the prediction class of the test sample is finally assigned to the class with the minimum reconstruction residual. Suppose that *n* samples of *m* dimensions construct a training matrix, $X\;\in \;R^{m\;\times \;n}$, where there are sufficient training samples belonging to the kth class, a submatrix constructed by sample of kth class samples can be symbolized as $X_{k}\;=\;\left[l_{k1},l_{k2}\ldots l_{kn_{k}}\right]$, where $l_{i}$ denotes the class of the *i*th sample and *n_k_* is the number of samples belonging to the kth class. Given a test sample $y\;\in \;R^{m}$, it can be represented as

(6) $$y=\alpha_{k,1}l_{k,1}+\alpha_{k,2}l_{k,2}+\cdots +\alpha_{k,n_{k}}l_{k,n_{k}}$$

When representing the whole training set, Equation (6) can be further transformed as

(7) $$y=X\alpha_{0}$$

where *α*_0_ = [0,…,0, *α*_k,1_, *α*_k,2_,.., 0,..,0]^T^ Since the nonzero entries in *α*_0_ are only associated with the *k*th class, *α*_0_ would come to be sparse when the class number of samples is large. In the SRC algorithm, the *α* vector, which can minimize the *l*_0_-norm of itself subject to Equation (7), is the key question that needs to be solved:

(8) $${\hat{\alpha }}_{0}=\arg\min||a|{|}_{0}\;\mathrm{subject}\;\mathrm{to}\;y=X\alpha$$

However, as an NP-hard problem, problem (8) can be achieved but it is hard to solve precisely. The theory of compressive sensing [32,33] shows that the *l*_0_-minimization problem can be solved by solving the solving the related convex *l*_1_-minimization problem instead when *α* is sparse enough:

(9) $${\hat{\alpha }}_{0}=\arg\min||a|{|}_{1}\;\mathrm{subject}\;\mathrm{to}\;y=X\alpha$$

Dealing with occlusion, we can extend Equation (9) to the stable *l*_1_-minimization problem:

(10) $${\hat{\alpha }}_{1}=\arg\min||a|{|}_{1}\;\mathrm{subject}\;\mathrm{to}\;||y-X\alpha ||\leq \varepsilon$$

where *ε* > 0 denotes the tolerance of the reconstruction error. Given the solution from Equation (10), the SRC algorithm assigns the label of test sample *y* to class *c* which has the minimum reconstruction residual:

(11) $$\underset{c}{\min}r_{c}(y)=\left\|y-X{\hat{\alpha }}_{1}^{c}\right\|,c=\;1,\;\ldots ,K$$

Besides sparse representation, Nearest Neighbor (NN) classifier is another popular classifier used in solving classification problems. Since the NN classifier only considers the nearest neighbor, it often easily suffers from the noise. However, research [34,35] has shown that locality is more essential than sparsity in some cases. Based on this theory, as a variant of conventional SRC, the weighted sparse representation-based classifier (WSRC) integrates the locality structure of data into basic sparse representation. Specifically, Gaussian distances between a sample and the whole set of training samples are first computed and used as the weights of each training sample. The Gaussian distance between two samples, *s*_1_ and *s*_2_, can be described as follows:

(12) $$d_{G}(s_{1},s_{2})=e^{-{\left\|s_{1}-s_{2}\right\|}^{2}/2\sigma^{2}}$$

where *d_G_* means the Gaussian kernel width. The information about the locality structure of the data can be retained by these weights. WSRC can then solve the following problem:

(13) $${\hat{\alpha }}_{1}=\arg\min||W\alpha |{|}_{1}\;\mathrm{subject}\;\mathrm{to}\;y=X\alpha$$

and specifically,

(14) $$diag(W)={\left[d_{G}(y,x_{1}^{1}),...,d_{G}(y,x_{n_{k}}^{k})\right]}^{T}$$

where *W* is a block-diagonal matrix of the locality adaptor and *n_k_* is the sample number of members in in class *k* in the training set. Dealing with occlusion, we can finally solve the following stable *l*_1_-minimization problem:

(15) $${\hat{\alpha }}_{1}=\arg\min||W\alpha |{|}_{1}\;\mathrm{subject}\;\mathrm{to}\;||y-X\alpha ||\leq \varepsilon$$

where *ε* > 0 is the tolerance value.

The WSRC algorithm can be summarized by the following steps:

**Algorithm 1. Weighted Sparse Representation-Based Classifier (WSRC)**

*1. Input: training samples matrix *
$X\in R^{m\times n}$
* and any test sample *
$y\in R^{d}$
*.*
*2. Normalize the columns of X to have unit l_2_-norm.*
*3. Calculate the Gaussian distances between y and each sample in X and make up matrix W.*
*4. Solve the stable l_1_-minimization problem defined in Eq.(13)*
*5. Compute each residual of K classes: *
$r_{c}(y)=||y-X{\hat{\alpha }}_{1}^{c}||$
* (c = 1, 2, …, K)*
*6. Ouput: assign y to class c by the rule: *
$identity(y)=\underset{c}{\arg\min}(r_{c}(y))$


### 4.5. Evaluation Measures

In order to measure the prediction performance of the proposed method, the overall prediction accuracy (Accu.), sensitivity (Sens.), precision (Prec.) and Matthews correlation coefficient (MCC) were calculated. They are defined as follows:

(16) $$\mathrm{Accu}.=\frac{\mathrm{TP}+\mathrm{TN}}{\mathrm{TP}+\mathrm{FP}+\mathrm{TN}+\mathrm{FN}}$$

(17) $$\mathrm{Sens}.=\frac{\mathrm{TP}}{\mathrm{TP}+\mathrm{FN}}$$

(18) $$\mathrm{Prec}.=\frac{\mathrm{TP}}{\mathrm{TP}+\mathrm{FP}}$$

(19) $$\mathrm{MCC}=\frac{\mathrm{TP}\times \mathrm{TN}-\mathrm{FP}\times \mathrm{FN}}{\sqrt{\left(\mathrm{TP}+\mathrm{FN})\times (\mathrm{TN}+\mathrm{FP})\times (\mathrm{TP}+\mathrm{FP})\times (\mathrm{TN}+\mathrm{FN}\right)}}$$

where the true positive (TP) value denotes the number of true samples which are predicted correctly; the false negative (FN) value is the number of true samples predicted to be non-interacting pairs incorrectly; the false positive (FP) value is the number of true non-interacting pairs predicted to be PPIs falsely; and the true negative (TN) value is the number of true non-interacting pairs predicted correctly. In addition, the receiver operating characteristic (ROC) curve was used to evaluate the performance of the proposed method. To summarize the ROC curve in a numerical way, the area under an ROC curve (AUC) was computed. 

### 4.6. Cross-Validation

As demonstrated in a series of studies [36,37,38,39,40,41,42,43], using three cross-validation methods, i.e., independent dataset test, K-fold cross-validation test and Leave-one-out cross-validation (LOOCV, also called jackknife cross validation), LOOCV is the most rigorous and objective evaluation method. However, to reduce the computational time, we adopted the five-fold cross-validation method in this study. In five-fold cross-validation, the training dataset is randomly divided into five parts, from which four parts were used for training, and the fifth part was used for testing. This process was repeated until all the parts were used at least once as a test set, and the overall performance on all five parts was evaluated.

## 5. Conclusions

In this post-genomic era, there has been great progress in regard to the computational methods applied to predict protein-protein interactions. In this work, we present a computational method only using the information from protein sequences to predict PPIs. This method is based on sparse representation and combines the continuous and discrete wavelet transforms. Specifically, all protein sequences are first transformed into numerical sequences based on the hydrophobicity index. These numerical sequences are then transformed into CW matrixes using the Meyer continuous wavelet transform and then the singular value decomposition method is used to extract CW-SVD features from the matrixes. In addition, DW features were also extracted from the discrete wavelet transform. These two complementary descriptors construct the whole feature space for protein sequence samples. The weighted sparse representation-based classifier was then used to deal with sample classification. Good results obtained from the experiments predicting PPIs of both one species data and cross-species data show that the proposed method has a great generalization ability and powerful ability to predict protein-protein interaction.

## Figures and Tables

**Table 1:** Five-fold cross validation result obtained in predicting the yeast PPIs dataset.

Test Set	Accuracy (%)	Precision (%)	Sensitivity (%)	MCC (%)	AUC (%)
1	97.32	100.00	94.70	94.77	97.69
2	97.63	100.00	95.24	95.37	97.67
3	97.05	100.00	94.08	94.26	97.05
4	97.76	100.00	95.63	95.63	97.55
5	97.14	100.00	94.13	94.43	97.41
Average	97.38 ± 0.31	100.00 ± 0.00	94.76 ± 0.68	94.89 ± 0.59	97.48 ± 0.26

**Table 2:** Five-fold cross validation result obtained in predicting the *H. pylori* PPIs dataset.

Test Set	Accuracy (%)	Precision (%)	Sensitivity (%)	MCC (%)	AUC (%)
1	92.28	96.72	88.04	85.72	92.72
2	93.83	95.94	91.23	88.38	93.74
3	94.68	95.99	92.93	89.91	94.20
4	95.20	97.70	93.40	90.81	95.36
5	93.66	95.70	90.41	88.01	94.99
Average	93.93 ± 1.11	96.41 ± 0.81	91.20 ± 2.15	88.57 ± 1.95	94.20 ± 1.05

**Table 3:** Five-fold cross validation result obtained in predicting the human PPIs dataset.

Classification Model	Testing Set	Accuracy (%)	Precision (%)	Sensitivity (%)	MCC (%)	AUC (%)
Proposed Method	1	99.19	99.86	98.38	98.39	99.41
	2	98.88	100.00	97.60	97.78	99.03
	3	98.70	99.87	97.47	97.43	98.78
	4	98.64	100.00	97.07	97.29	98.41
	5	99.20	100.00	98.33	98.40	99.03
	Average	98.92 ± 0.27	99.95 ± 0.07	97.77 ± 0.57	97.86 ± 0.52	98.93 ± 0.37
Combined Wavelet Feature with SVM	1	91.39	96.15	84.57	83.91	94.71
	2	90.77	97.17	82.53	82.83	94.77
	3	88.85	96.08	80.53	79.87	93.65
	4	88.79	94.53	80.56	79.62	91.50
	5	90.85	96.75	83.85	83.14	95.33
	Average	90.13 ± 1.22	96.14 ± 1.00	82.41 ± 1.85	81.87 ± 1.99	93.99 ± 1.52

**Table 4:** Performance comparison of different methods on the yeast dataset. (N/A means Not applicable).

Method	Approach	Accuracy (%)	Precision (%)	Sensitivity (%)	MCC (%)
Guos’ work [18]	ACC	89.33 ± 2.67	88.87 ± 6.16	89.93 ± 3.68	N/A
	AC (Auto Covariance)	87.36 ± 1.38	87.82 ± 4.33	87.30 ± 4.68	N/A
Zhous’ work [19]	SVM + LD	88.56 ± 0.33	89.50 ± 0.60	87.37 ± 0.22	77.15 ± 0.68
Yangs’ work [20]	Cod1	75.08 ± 1.13	74.75 ± 1.23	75.81 ± 1.20	N/A
	Cod2	80.04 ± 1.06	82.17 ± 1.35	76.77 ± 0.69	N/A
	Cod3	80.41 ± 0.47	81.86 ± 0.99	78.14 ± 0.90	N/A
	Cod4	86.15 ± 1.17	90.24 ± 1.34	81.03 ± 1.74	N/A
Huangs’ work [21]	CW + PseAAC	92.05 ± 0.59	95.87 ± 0.89	88.82 ± 0.98	86.09 ± 1.02
Our work	WSRC + AM [22]	96.03 ± 0.55	100.00 ± 0.00	92.07 ± 1.03	92.36 ± 1.01
	WSRC + BGR [22]	96.14 ± 0.43	100.00 ± 0.00	92.29 ± 0.77	92.55 ± 0.80
	WSRC + LBP − HF [23]	96.60 ± 0.31	100.00 ± 0.00	93.20 ± 0.69	93.42 ± 0.58
	WSRC+ LPQ [23]	96.25 ± 0.17	100.00 ± 0.00	92.51 ± 0.45	92.77 ± 0.33
	WSRC + CW&DW	97.38 ± 0.31	100.00 ± 0.00	94.76 ± 0.68	94.89 ± 0.59

**Table 5:** Performance comparison of different methods on the *H. pylori* dataset. (N/A means Not applicable).

Method	Accuracy (%)	Precision (%)	Sensitivity (%)	MCC (%)
Phylogenetic Booststrap [24]	75.80	80.20	69.80	N/A
HKNN [25]	84.00	84.00	86.00	N/A
Signature Products [26]	83.40	85.70	79.90	N/A
Ensemble of HKNN [27]	86.60	85.00	86.70	N/A
Boosting [28]	79.52	81.69	80.37	70.64
Proposed Method	93.93	96.41	91.20	88.57

**Table 6:** Prediction results for six species based on our model.

Species	Test Pairs	Accuracy
*D. mela*	21774	97.36%
*E. coli*	6897	86.56%
*C. elegans*	4013	96.64%
*H. sapien*	1406	94.24%
*H. pylori*	1420	95.07%
*M. musculus*	312	94.23%

## References

[1] Walsh I., Di Domenico T., Tosatto S.C. (2014). RUBI: Rapid proteomic-scale prediction of lysine ubiquitination and factors influencing predictor performance. Amino Acids.

[2] Ito T., Chiba T., Ozawa R., Yoshida M., Hattori M., Sakaki Y. (2001). A comprehensive two-hybrid analysis to explore the yeast protein interactome. Proc. Nat. Acad. Sci. USA.

[3] Pazos F., Valencia A. (2002). In silico two-hybrid system for the selection of physically interacting protein pairs. Proteins Struct. Funct. Bioinform..

[4] Gavin A.-C., Bösche M., Krause R., Grandi P., Marzioch M., Bauer A., Schultz J., Rick J.M., Michon A.-M., Cruciat C.-M. (2002). Functional organization of the yeast proteome by systematic analysis of protein complexes. Nature.

[5] Ho Y., Gruhler A., Heilbut A., Bader G.D., Moore L., Adams S.-L., Millar A., Taylor P., Bennett K., Boutilier K. (2002). Systematic identification of protein complexes in Saccharomyces cerevisiae by mass spectrometry. Nature.

[6] Zanzoni A., Montecchi-Palazzi L., Quondam M., Ausiello G., Helmer-Citterich M., Cesareni G. (2002). MINT: A Molecular INTeraction database. FEBS Lett..

[7] Xenarios I., Rice D.W., Salwinski L., Baron M.K., Marcotte E.M., Eisenberg D. (2000). DIP: The database of interacting proteins. Nucleic Acids Res..

[8] Agrawal N.J., Helk B., Trout B.L. (2014). A computational tool to predict the evolutionarily conserved protein-protein interaction hot-spot residues from the structure of the unbound protein. FEBS Lett..

[9] Qiu Z., Wang X. (2012). Prediction of protein-protein interaction sites using patch-based residue characterization. J. Theor. Biol..

[10] Tosatto S., Toppo S. (2006). Large-scale prediction of protein structure and function from sequence. Curr. Pharm. Des..

[11] Giollo M., Minervini G., Scalzotto M., Leonardi E., Ferrari C., Tosatto S.C. (2015). BOOGIE: Predicting blood groups from high throughput sequencing data. PLoS ONE.

[12] Zahiri J., Yaghoubi O., Mohammad-Noori M., Ebrahimpour R., Masoudi-Nejad A. (2013). PPIevo: Protein-protein interaction prediction from PSSM based evolutionary information. Genomics.

[13] Chanphai P., Bekale L., Tajmir-Riahi H. (2015). Effect of hydrophobicity on protein-protein interactions. Eur. Polym. J..

[14] Koike A., Takagi T. (2004). Prediction of protein-protein interaction sites using support vector machines. Protein Eng. Des. Sel..

[15] Dong Q., Wang X., Lin L., Guan Y. (2007). Exploiting residue-level and profile-level interface propensities for usage in binding sites prediction of proteins. BMC Bioinform..

[16] Cai L., Pei Z., Qin S., Zhao X. Prediction of protein-protein interactions in saccharomyces cerevisiae based on protein secondary structure. Proceedings of the 2012 IEEE International Conference on Biomedical Engineering and Biotechnology (iCBEB).

[17] Chen H., Zhou H.X. (2005). Prediction of interface residues in protein-protein complexes by a consensus neural network method: Test against NMR data. Proteins Struct. Funct. Bioinform..

[18] Guo Y., Yu L., Wen Z., Li M. (2008). Using support vector machine combined with auto covariance to predict protein-protein interactions from protein sequences. Nucleic Acids Res..

[19] Zhou Y.Z., Gao Y., Zheng Y.Y. (2011). Prediction of Protein-Protein Interactions Using Local Description of Amino Acid Sequence. Advances in Computer Science and Education Applications.

[20] Yang L., Xia J.-F., Gui J. (2010). Prediction of protein-protein interactions from protein sequence using local descriptors. Protein Pept. Lett..

[21] Huang Y.-A., You Z.-H., Chen X., Yan G.-Y. (2016). Improved protein-protein interactions prediction via weighted sparse representation model combining continuous wavelet descriptor and PseAA composition. BMC Syst. Biol..

[22] Nanni L., Lumini A., Brahnam S. (2014). An empirical study of different approaches for protein classification. Sci. World J..

[23] Nanni L., Brahnam S., Lumini A. (2012). Wavelet images and Chou’s pseudo amino acid composition for protein classification. Amino Acids.

[24] Bock J.R., Gough D.A. (2003). Whole-proteome interaction mining. Bioinformatics.

[25] Nanni L. (2005). Hyperplanes for predicting protein-protein interactions. Neurocomputing.

[26] Martin S., Roe D., Faulon J.-L. (2005). Predicting protein-protein interactions using signature products. Bioinformatics.

[27] Nanni L., Lumini A. (2006). An ensemble of K-local hyperplanes for predicting protein-protein interactions. Bioinformatics.

[28] Shi M.-G., Xia J.-F., Li X.-L., Huang D.-S. (2010). Predicting protein-protein interactions from sequence using correlation coefficient and high-quality interaction dataset. Amino Acids.

[29] You Z.-H., Yu J.-Z., Zhu L., Li S., Wen Z.-K. (2014). A MapReduce based parallel SVM for large-scale predicting protein-protein interactions. Neurocomputing.

[30] Li F.M., Li Q.Z. (2008). Predicting Protein Subcellular Location Using Chous Pseudo Amino Acid Composition and Improved Hybrid Approach. Protein Pept. Lett..

[31] Wright J., Ganesh A., Zhou Z., Wagner A., Ma Y. Demo: Robust Face Recognition Via Sparse Representation. Proceedings of the 8th IEEE International Conference on Automatic Face & Gesture Recognition.

[32] Candes E., Tao T. (2004). Near Optimal Signal Recovery From Random Projections: Universal Encoding Strategies?. IEEE Trans. Inf. Theory.

[33] Chen S.S., Donoho D.L., Saunders M.A. (2001). Atomic decomposition by basis pursuit. SIAM Rev..

[34] Roweis S.T., Saul L.K. (2000). Nonlinear Dimensionality Reduction by Locally Linear Embedding. Science.

[35] Wang J., Yang J., Yu K., Lv F., Huang T., Gong Y. Locality-constrained Linear Coding for image classification. Proceedings of the 2010 IEEE Conference on Computer Vision and Pattern Recognition (CVPR).

[36] Feng P.-M., Chen W., Lin H., Chou K.-C. (2013). iHSP-PseRAAAC: Identifying the heat shock protein families using pseudo reduced amino acid alphabet composition. Anal. Biochem..

[37] Lynch C.M., van Berkel V.H., Frieboes H.B. (2017). Application of unsupervised analysis techniques to lung cancer patient data. PLoS ONE.

[38] Manavalan B., Shin T.H., Lee G. (2018). DHSpred: Support-vector-machine-based human DNase I hypersensitive sites prediction using the optimal features selected by random forest. Oncotarget.

[39] Manavalan B., Basith S., Shin T.H., Choi S., Kim M.O., Lee G. (2017). MLACP: Machine-learning-based prediction of anticancer peptides. Oncotarget.

[40] Manavalan B., Lee J. (2017). SVMQA: Support–vector-machine-based protein single-model quality assessment. Bioinformatics.

[41] Manavalan B., Lee J., Lee J. (2014). Random forest-based protein model quality assessment (RFMQA) using structural features and potential energy terms. PLoS ONE.

[42] Feng P., Yang H., Ding H., Lin H., Chen W., Chou K.-C. (2018). iDNA6mA-PseKNC: Identifying DNA N6-methyladenosine sites by incorporating nucleotide physicochemical properties into PseKNC. Genomics.

[43] Chen W., Yang H., Feng P., Ding H., Lin H. (2017). iDNA4mC: Identifying DNA N4-methylcytosine sites based on nucleotide chemical properties. Bioinformatics.

